# A pilot study to determine the optimal dose of scAAVIL-1ra in a large animal model of post-traumatic osteoarthritis

**DOI:** 10.1038/s41434-023-00420-2

**Published:** 2023-09-11

**Authors:** P. Thampi, K. A. Seabaugh, L. M. Pezzanite, C. R. Chu, J. N. Phillips, J. C. Grieger, C. W. McIlwraith, R. J. Samulski, L. R. Goodrich

**Affiliations:** 1 Orthopaedic Research Center, C. Wayne McIlwraith Translational Medicine Institute, Colorado State University, Fort Collins, CO USA; 2 Department of Orthopaedic Surgery, Stanford University, Stanford, CA USA; 3 Veterans Affairs Palo Alto Healthcare System, Palo Alto, CA USA; 4 Gene Therapy Center, University of North Carolina, Chapel Hill, NC USA

**Keywords:** Targeted gene repair, Drug delivery

## Abstract

Gene therapy approaches using adeno-associated viral vectors have been successfully tested in the equine post-traumatic osteoarthritis (PTOA) model. Owing to differences in the levels of transgene expression and adverse tissue reactions observed in published studies, we sought to identify a safe therapeutic dose of scAAVIL-1ra in an inflamed and injured joint that would result in improved functional outcomes without any adverse events. scAAVIL-1ra was delivered intra-articularly over a 100-fold range, and horses were evaluated throughout and at the end of the 10-week study. A dose-related increase in IL-1ra levels with a decrease in PGE_2_ levels was observed, with the peak IL-1ra concentration being observed 7 days post-treatment in all groups. Perivascular infiltration with mononuclear cells was observed within the synovial membrane of the joint treated with the highest viral dose of 5 × 10^12^ vg, but this was absent in the lower-dosed joints. The second-highest dose of scAAVeqIL-1ra 5 × 10^11^ vg demonstrated elevated IL-1ra levels without any cellular response in the synovium. Taken together, the data suggest that the 10-fold lower dose of 5 × 10^11^vg scAAVIL-1ra would be a safe therapeutic dose in an equine model of PTOA.

## Introduction

Post-traumatic osteoarthritis (PTOA) is a leading cause of disability in humans with substantial associated economic burden and diminished quality of life [1, 2]. Although commonly associated with age and obesity, PTOA can also occur in younger individuals, particularly after acute or repetitive joint injuries. Osteoarthritis is also a significant clinical problem in the equine athlete with PTOA-associated lameness being the primary factor contributing to diminished athletic function and loss of performance [3–5]. Existing pharmacological and surgical methods to treat PTOA usually fail to restore normal articular cartilage structure and function, and, therefore, a long-term permanent cure is actively being explored [4, 6–10]. While equine studies of PTOA directly impact veterinary care, horses are also an important translational large animal model for evaluating new joint therapies intended for humans [4, 11–16]. Thus, preclinical studies in horses can be advantageous in advancing the field of musculoskeletal regenerative medicine for both human and equine PTOA.

While cartilage damage and the associated cellular changes are known to be central to the pathophysiology of PTOA, the role of inflammation in the initiation and progression of PTOA has been recognized for 45 years in the equine literature [17–22]. The most important among the pro-inflammatory triggers involved in PTOA is interleukin-1β (IL-1β). An IL-1β mediated inflammatory cascade leads to the downstream production of catabolic cytokines, chemokines, and neuropeptides such as IL-6, cyclooxygenases I and II, nitric oxide, phospholipase A2, prostaglandin E2, reactive oxygen species, which are potentially responsible for ECM degradation and joint pain [23, 24]. Because of the above effects, IL-1β offers a potential target for dampening this inflammatory cascade using the natural antagonist of IL-1β, Interleukin-1 receptor antagonist (IL-1ra). IL-1ra has been used clinically as a recombinant protein Anakinra/Kineret® in human and animal patients; however, the efficacy is limited due to the short half-life of the protein [25–28].

In order to extend the availability of the delivered protein in joint tissues, viral vector-based gene delivery systems have been extensively investigated. In early studies, first-generation adenoviral vectors were used to deliver IL-1ra directly into healthy equine joints, which resulted in elevated intra-articular expression of IL-1ra for approximately 28 days. The therapeutic IL-1ra levels were reflected in a significant improvement in clinical parameters of pain and degree of lameness, and beneficial effects on the synovial membrane and articular cartilage histologically. However, administration of the adenoviral vector was associated with mild-moderate perivascular lymphocytic infiltration [29, 30]. A significant improvement in the field was the discovery of self-complementary adeno-associated viruses (scAAVs) which demonstrated greater transduction efficiencies and prolonged transgene expression compared to adenoviral and other viral vectors [31, 32]. Since then, scAAV vectors have been tested extensively in several small animal and equine models [33–43]. A dosing trial conducted by our group delivering scAAVIL-1ra into normal equine joints demonstrated that therapeutic levels of IL-1ra could be detected up to 273 days following intra-articular injection [34]. In the same study, we observed that both in situ synoviocytes and chondrocytes could be efficiently transduced with intra articular injection of scAAVGFP for up to 4 months following injection. This is a significant advantage over most adenoviral mediated gene delivery studies where therapeutic levels can be detected only up to 1 month post-injection [30, 44]. In our study, delivering different doses of scAAVIL-1ra into healthy equine joints, the highest dose of 5 × 10^12^ vg scAAVIL-1ra tested provided sustained therapeutic levels of IL-1Ra following injection without any adverse effects. This was consistent with the observations of Watson Levings et al. [38] where the same dose of 5 × 10^12^ vg was identified as a safe therapeutic dose in healthy equine joints with no adverse tissue reactions. Further, another study by this group where the same dose of 5 × 10^12^ vg of scAAVIL-1ra was administered into joints of an induced equine PTOA model, resulted in significantly higher synovial fluid IL-1ra levels with an improved functional outcome of reduction in lameness compared to uninjured joints [39]. No adverse effects were observed at this 5 × 10^12^ vg dose in this induced PTOA model. In contrast to the observations from these studies, we have recently completed a study where the same dose of 5 × 10^12^ vg of scAAVIL-1ra generated perivascular cuffing within the synovial membrane of joints in an equine model of PTOA [33].

Due to these conflicting observations, we proposed to optimize the therapeutic dose of scAAVIL-1ra in an well-validated experimental joint model of equine PTOA. Our objective was to examine if administration of scAAVIL-1ra in this model will lead to improved functional outcomes, with the long-term goal of further testing in Phase I preclinical studies in horses. We postulated that scaled doses of scAAVIL-1ra administration in an equine model of PTOA would be reflected in proportional levels of IL-1ra protein in the synovial fluid of injected joints. In addition, we sought to determine if an optimized dose would provide therapeutic levels of protein intraarticularly and be associated with an improvement in biochemical, gross, and histological outcomes without a negative outcome in the synovial tissue.

## Materials and Methods

### Experimental Design

**Fig. 1:**
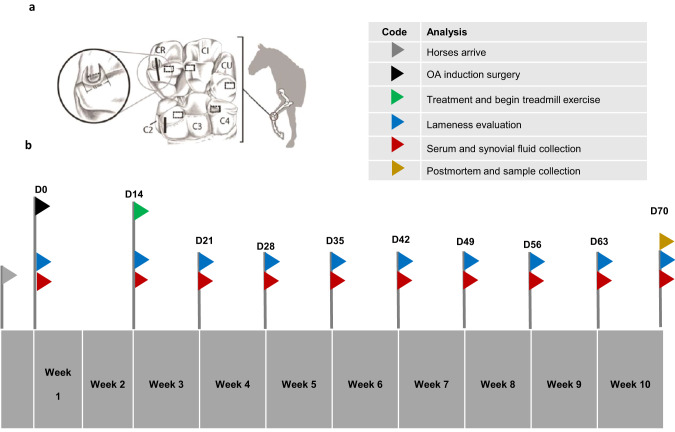
Equine middle carpal joint illustration and study timeline. An illustration (**a**) of the equine middle carpal joint indicating the area where the osteochondral chip fragment was induced (close-up view shown in inset circle). Rectangular boxes indicate areas from which samples were taken for cartilage histology. The solid vertical line indicates areas in which osteochondral blocks were harvested for histological assessment of cartilage and subchondral bone. Diagram modified from Frisbie et al. Gene Therapy, 2002 with permission. **b** The study timeline with color-coded flags illustrating the time points at which treatments were administered, lameness evaluations, and sample collections were performed.

Five skeletally mature horses aged 2–5 years with no radiographic abnormalities within the carpus nor signs of lameness were approved by the Institutional Animal Care and Use Committee (IACUC number -1305) for use in this study. All horses underwent bilateral arthroscopic surgery of the middle carpal joints (Fig. 1a) (following anesthesia as described previously [5]. An osteochondral defect was created in one randomly selected middle carpal joint, followed by defect enlargement using a motorized arthroburr which liberates cartilage and bone debris within the synovial cavity [30]. The contralateral joint served as the sham-operated control undergoing arthroscopy only. Fourteen days after fragment creation, one horse each received one of the following doses of scAAVIL-1ra vectors brought to a final volume of 5 ml in saline; 5 × 10^12^vg, 5 × 10^11^vg, 1 × 10^11^vg, 5 × 10^10^vg and 1 × 10^10^ vg. The control joints received the same volume of 0.9% saline intra-articularly (IA). Horses were housed in stalls (3.65 m x 3.65 m) and began a strenuous exercise regimen on a high-speed treadmill at 14 days post-surgery. Each horse was trotted for 2 min (16–19 km/h), galloped for 2 min (32 km/h) and trotted for 2 min (16–19 km/h), 5 days per week until the end of the study.

### Synovial fluid and serum evaluation

Synovial fluid was collected at the time of surgery (day 0), day 14, and weekly after day 14 until the termination of the study at 10 weeks (Fig. 1b). An aliquot of synovial fluid was assessed immediately for color, clarity, total protein, and fluid cytology using routine clinicopathologic parameters. Remaining synovial fluid aliquots were stored at –80 °C until the end of the study when they were analyzed for the presence of Interleukin-1-receptor antagonist (IL-1ra), Interleukin-1β (IL-1β), Prostaglandin E_2_ (PGE_2_), and Glycosaminoglycan (GAG). Serum was collected from all horses and assessed for the presence of IL-1ra and GAG. Correlations between IL-1ra and PGE_2_ were determined using a Pearson’s correlation test.

### Gross pathological examination

Post euthanasia, forelimbs were removed mid-radius and aseptically prepped for gross examination. Middle carpal joints were disarticulated and subjectively scored on a scale of full and partial thickness cartilage erosions, hemorrhage and synovial adhesions. Articular cartilage samples were collected from areas of the joint depicted in Fig. 1a. Osteochondral sections were taken from the radial and third carpal bones for Hematoxylin and Eosin (H&E) and Safranin-O/Fast Green (SOFG) staining. Synovial membrane samples were taken from the area closest to the defect and were assessed with H&E staining.

### Histological examination

Synovial membrane, articular cartilage and osteochondral samples were placed in 10% neutral buffered formalin solution for 48 h. Osteochondral samples were decalcified in Formical solution (American MasterTech; Lodi, CA) and monitored daily until decalcification was complete based on the pliability of samples. All samples were stained with either Harris Hematoxylin for 10 min and Eosin Y counterstain, or Weigert’s Hematoxylin and 0.1% aqueous Safranin O for 7 min and 0.2% aqueous Fast Green counterstain. Equine trachea was used as a positive control.

H&E stained synovial membrane sections were blindly evaluated for cellular infiltration, intimal hyperplasia, subintimal edema, subintimal fibrosis and vascularity as previously described [45]. H&E stained articular cartilage sections were evaluated for chondrocyte necrosis, chondrone formation, fibrillation, and focal cell loss. All assessments were made using a 5-point scale with 0 indicating no change in the tissue and 4 indicating severe changes, with total summary scores being the sum of all four categories (total cumulative score ranging from 0 to 16). SOFG stained articular cartilage sections were blindly evaluated based on the uptake of stain by the tissue using an ordinal scale with 0 indicating normal stain uptake and 4 indicating uptake of 25% or less of the normal stain.

### Clinical outcomes

Clinical examinations of both forelimbs were made by a board certified specialist of the American College of Veterinary Sports Medicine and Rehabilitation (ACVSMR) at day 0, day 10 and then weekly until the end of the study period. Lameness (pain) was graded on a standardized 0-5 scale [46], response to carpal flexion and carpal effusion on a 0-4 scale [47, 48] and range of motion on a 0-3 scale (0-normal, 3-severe).

## Results

In this pilot study, PTOA was induced in one joint of each horse and the contralateral joint was sham operated and served as the control, non-PTOA joint. All PTOA joints received scAAVIL-1ra at different doses intra-articularly while the non-PTOA joints received saline. Induction of PTOA in this model was validated by consistent clinical outcomes, synovial fluid analyses, postmortem examinations and histologic evaluation of synovial membrane, cartilage, and subchondral bone. The treatment group in the following sections refers to scAAVIL-1ra treated PTOA joints and the untreated group refers to sham operated (saline-injected) joints.

### Synovial fluid and serum evaluation

**Fig. 2:**
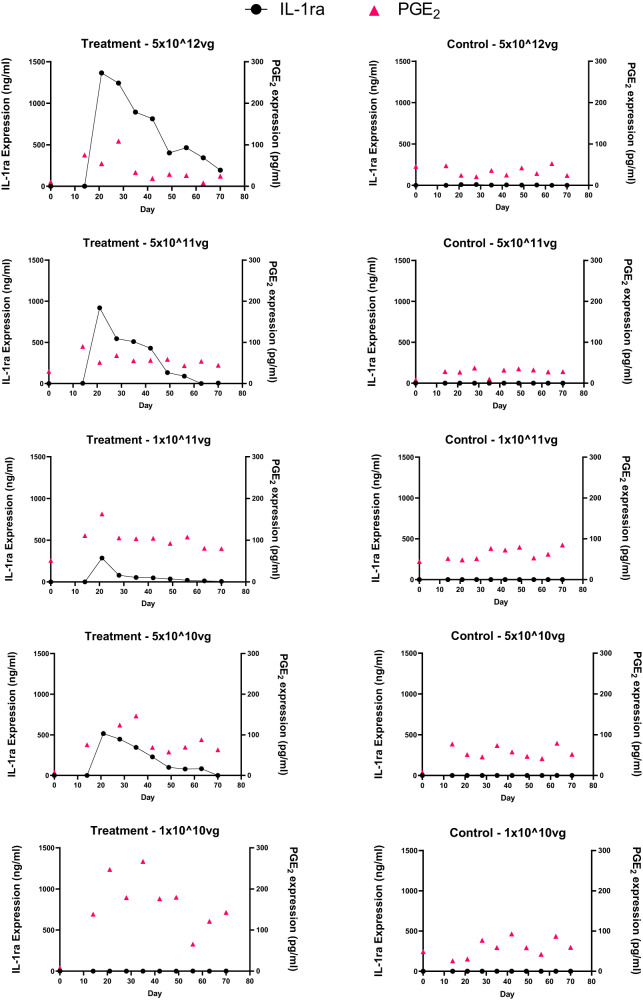
Mean IL-1ra (ng ml^−1^) (left axis) and mean PGE_2_ (pg ml^−1^) (right axis) levels in all five doses tested (left panel) along with the corresponding sham-operated control limb synovial fluid samples (right panel). Synovial fluid IL-1ra levels (black circles) increased with increasing viral dose, and PGE_2_ levels (red triangles) showed a decrease with increasing viral dose. IL-1ra levels tapered off to baseline towards the end of the study in all dosed OA joints. IL-1ra and PGE_2_ levels were not different between sham-operated control joints throughout the study period.

Mean IL-1ra levels in the synovial fluid were greatest at the two highest doses tested over the course of the study (Fig. 2; left Y-axis; Supplemental Table 1). Treatment with intra-articular scAAVIL-1ra at the highest dose (5 × 10^12^vg per joint) and second highest dose (5 × 10^11^vg per joint) resulted in peak levels (at 7 days) of IL-1ra protein of approximately 1300 ng/ml and 900 ng/ml respectively compared to untreated joints. At this highest dose, the mean IL-1ra concentration within the treated joints remained over 150 fold higher than the mean concentration in the untreated joints (572.02 ± 21.48 ng/ml in the treated joints vs 3.71 ± 1.82 ng/ml in the untreated joints). At the second highest dose of 5 × 10^11^vg per joint, the mean IL-1ra concentration was over 800 fold higher in the treated joints compared to the control joints throughout the study period (263.88 ± 17.41 ng/ml in the treated joints vs 0.33 ± 0.29 ng/ml in the untreated joints). The IL-1ra levels peaked at 7 days after administration of the vector (Day 21) and gradually tapered off to baseline by the end of the study. This trend in peak IL-1ra protein levels was observed in all the doses tested except the lowest dosed joint (1 × 10^10^vg per joint). The lowest dosed joint showed low levels of IL-1ra which were comparable to the levels in sham operated controls (0.47 ± 0.34 ng/ml in the treated joints vs 0.31 ± 0.04 ng/ml in the untreated joints).

Mean PGE_2_ levels showed a decline in the treated joints with increasing viral dose (Fig. 2; right Y-axis; Supplemental Table 1). The mean concentration of PGE_2_ in the lowest dose group (1 × 10^10^vg) was almost 4-fold higher (152.54 ± 8.56 pg/ml) than the group dosed with the highest dose of 5 × 10^12^vg (38.88 ± 5.50 pg/ml). Similar to IL-1ra, PGE_2_ levels peaked at Day 21 with a 5-fold increase from the highest to the lowest doses tested (54 pg/ml in the highest dose vs 247 pg/ml in the lowest dose). There were no significant changes in other biochemical markers tested and in fluid cytology parameters tested in the synovial fluid or serum (data not shown). Correlation analysis was perfomed between the mean IL-1ra and PGE_2_ expression levels at each viral dose using Pearson’s correlation. No associations were observed between IL-1ra and PGE_2_ levels in the treated horses at 3 of the 5 doses tested. IL-1ra and PGE_2_ levels showed a significant correlation at two doses (1 × 10^11^ vg and 5 × 10^10^ vg) with an R value 0.8333 and 0.7364. Correlation analysis was also performed between the viral dose and the peak IL-1ra or PGE_2_ levels. The peak IL-1ra and PGE_2_ levels at day 21 were weakly inversely correlated with viral dose in all treated horses (Supplementary Tables 2, 3).

### Gross pathological examination

**Fig. 3:**
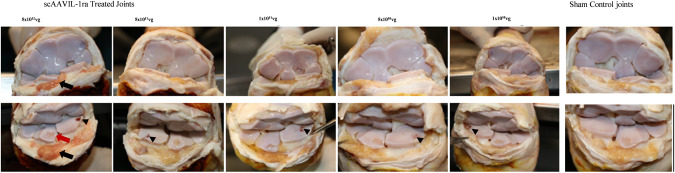
Gross images highlighting the osteochondral chip fragment on the radial carpal bone (black arrowhead), hemorrhagic synovium (black arrow) and site of aseptic cartilage harvest on the intermediate carpal bone (red arrow) from all doses tested. Representative photographs from a sham operated control joint which received saline is shown on the right panel. Top panel shows the distal view, and the bottom panel shows the proximal view of the carpal joint.

All joints with an osteochondral chip fragment resulted in some level of pathologic change compared to the sham operated controls, confirming that induction of PTOA was successful in this model (Fig. 3). All PTOA induced joints demonstrated kissing lesions on the opposing surface of the joint where the fragments were created. Synovial adhesions were present only in the lowest dosed joint (1 × 10^10^vg per joint). The site of fragment creation showed varying degrees of hemorrhage and erosions compared to the untreated joints, however, the erosion scores were not different between the different doses tested (data not shown). Gross hemorrhage was markedly higher in the highest dosed joint with moderate to low hemorrhage in the lower dosed joints.

### Histological evaluation

**Fig. 4:**
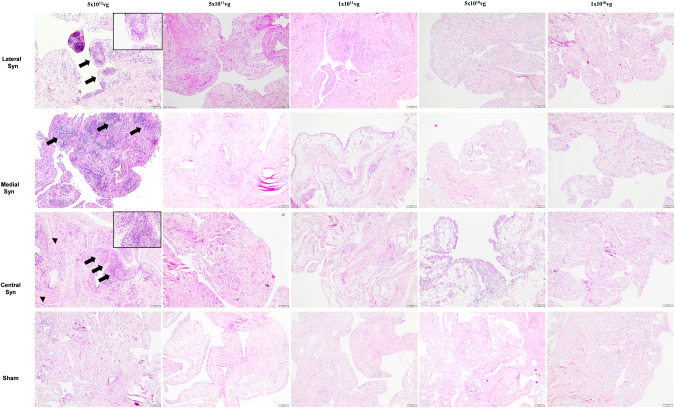
Photomicrograph from 5 μm sections of synovial membrane (lateral, medial, central) stained with H&E from horses treated with different doses of viral vectors with the corresponding sham control joints (bottom panel). The synovial membrane from the horse administered the highest viral dose (5 x 10^12^vp scAAVIL-1ra) showed perivascular lymphocytic infiltration (black arrows; inset) and subintimal fibrosis (black arrowheads). Sections of synovial membrane from lower dosed joints lack any adverse tissue reactions. Plates are at 10X and insets are at 40X magnification.

**Fig. 5:**
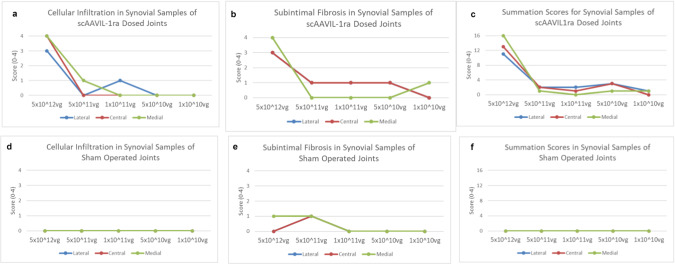
Effects of scAAVIL-1ra gene transfer on the synovial membrane. Cellular infiltration (**a**), subintimal fibrosis (**b**), and summation scores (**c**) in OA joints compared to sham operated joints (**d**–**f**). All scores are significantly higher in the highest viral dose tested in agreement with the qualitative histological analysis.

Histological examination of H&E stained synovial membrane from the treated joints revealed perivascular cuffing and subintimal fibrosis in the highest dosed joint (Fig. 4). Consistent with this observation, cellular infiltration and subintimal fibrosis scores were worse in the highest dosed joint compared to the second highest dosed joint and all other dosed joints tested, contributing to an increase (worsening) in overall scores in this group (Fig. 5). All joints other than the joint injected with the highest (5 x 10^12^) dose of scAAVIL-1ra showed histological appearance and scores comparable to the untreated controls. Synovial membrane summation scores (cellular infiltration, intimal hyperplasia, subintimal edema, subintimal fibrosis and vascularity) were higher overall for PTOA joints treated with scAAVIL-1ra compared with sham operated control joints.

**Fig. 6:**
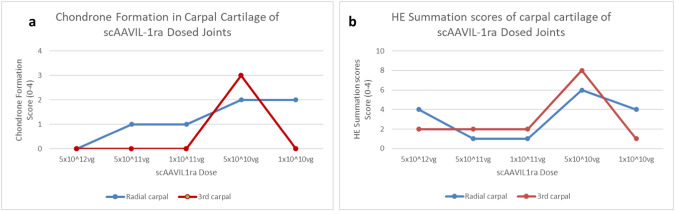
Effects of scAAVIL-1ra viral gene transfer on the cartilage. Chondrone formation (**a**) and HE summation scores (**b**) in OA joints. The joint dosed with 5x10^10^vp scAAVIL-1ra showed high chondrone formation compared to other treatment groups.

**Fig. 7:**
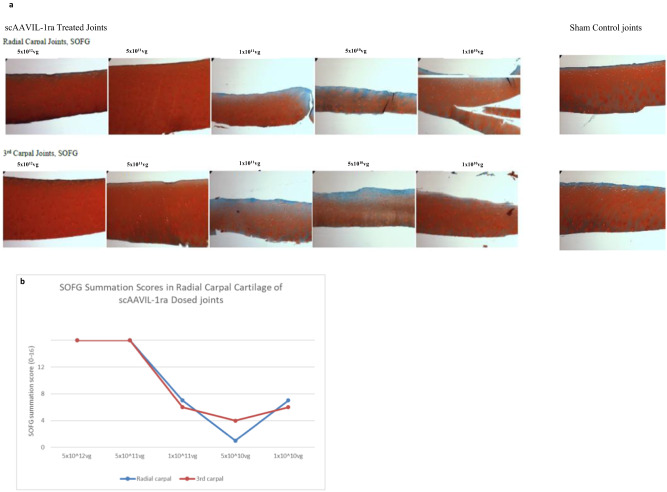
Effects of viral gene transfer on cartilage quality. **a** Photomicrograph from 5-μm sections of cartilage from radial and 3rd carpal bone stained with SOFG. The left panel represents cartilage from joints treated with different doses of viral vectors and the right panel shows a representative section from the control joints. **b** SOFG stain uptake scores in radial and 3rd carpal joints. The top two doses tested showed higher stain uptake compared to the lower doses.

Assessment of the articular cartilage from the weight bearing surfaces of the radial and 3rd carpal bone confirmed the success of PTOA induction in this model. The cartilage from both locations showed higher (worsening) summation scores (cartilage fibrillation, chondrone formation, chondrocyte necrosis and focal cell loss) with a decrease in viral dose (Fig. 6). The greatest treatment effects were observed for chondrone formation subscores. In addition, SOFG stain uptake, an indicator of proteoglycan content, showed a proportionate decrease with decreasing viral dose. This was also reflected in the SOFG summation scores with the highest scores (improvement) observed in the highest dosed joint (Fig. 7).

### Clinical outcomes

Based on outcome assessments starting 14 days after PTOA induction, expected changes in lameness, pain response to standing flexion, lameness following joint flexion, decrease in degrees of flexion, and effusion were observed in all horses consistent with the successful induction of PTOA.

## Discussion

We report the results of an in vivo dosing study to determine the effect of vector dose on eq-IL-1ra expression following intra-articular delivery of scAAVIL-1ra over a 100-fold range (from 5×10^12^ to 1x10^10^ vg) in a well-established surgically induced model of PTOA. This vector has been tested by our group and others in several studies and shown to result in stable and persistent production of IL-1ra protein intra-articularly with no deleterious effects [34, 35, 38, 40].

Consistent with findings from our previous studies, treatment with scAAVIL-1ra resulted in elevated and therapeutic synovial fluid IL-1ra levels in all treated joints throughout the study. An important finding in this study was the perivascular cellular infiltration in the synovium along with subintimal fibrosis in the highest viral dose tested (5 x 10^12^vg). It is worth noting that perivascular cuffing and subintimal hyperplasia has been observed using adenovirus vectors [29, 30, 44] but adverse tissue reactions have not been reported previously in the synovium using scAAV vectors in healthy joints [34, 35, 38, 40] nor in an induced PTOA model at the same viral dose of 5 x 10^12^vg in a study performed by another group [39]. However, we observed an adverse tissue reaction in our equine induced PTOA model [33] which prompted us to undertake the current study to optimize the therapeutic viral dose in the same model. Consistent with our previous findings [33], in the present study we observed a cellular reaction in the synovium at the viral dose of 5 x 10^12^ vg. Although this adverse tissue reaction did not negatively affect the IL-1ra levels in the synovial fluid, the authors feel that this is an undesirable outcome and a 10-fold lower dose (5 x 10^11^vg) is optimal as it results in therapeutic levels of IL-1ra but also minimizes the risk of local tissue reactions to viral vector administration.

The mean concentration of IL-1ra in the synovial fluid of all treated joints was substantially higher compared to untreated joints in our study. Mean IL-1ra levels in synovial fluid from the sham-operated joints remained at pretreatment levels ( <1 ng/mL) throughout. Interestingly, the peak IL-1ra levels at the 5 x 10^12^ vg dose observed in this study ( >1300 ng/ml) was more than 2-fold higher and 20-fold higher than the peak IL-1ra concentration in healthy equine joints in studies conducted by our group (500 ng/ml) [34] and others (50 ng/ml) [38] respectively (Table 1). Consistent with our observations, Watson-Levings et al. also reported approximately four fold higher IL-1ra levels in PTOA joints (214 ng/ml) compared to healthy non-PTOA joints (59 ng/ml) [39]. The cytomegalovirus (CMV) promoter, which drives the expression of the scAAV cassette, is known to be activated by inflammatory mediators and stress-activated chemokines [49–52]. As PTOA-cartilage is enriched in stress-activated chondrocytes, particularly at the site of injury, we postulate that the drastic upregulation in transgene expression that we observed in an injured joint is a result of inflammation-induced activation of the CMV promoter in response to mediators in the diseased joint environment.

**Table 1:** Comparison of synovial fluid IL-1ra levels from normal and induced OA joints from published studies using the scAAV vector.

Study	Equine model	Viral vector used	Duration of study	Sample size	Viral dose	Mean IL-1ra concentration in synovial fluid
Goodrich, 2013 [35]	Normal joints	scAAVIL-1ra	23 days	*n* = 1	5 × 10^12^ vg	200–1600 ng/ml
			28 weeks	*n* = 1	5 × 10^13^ vg	200–1800 ng/ml
Goodrich, 2015 [34]	Normal joints	scAAVIL-1ra	39 weeks	*n* = 2	5 × 10^12^ vg	200–500 ng/ml
				*n* = 2	5 × 10^11^ vg	100–300 ng/ml
				*n* = 2	5 × 10^10^ vg	10–80 ng/ml
Watson-Levings, 2018 [38]	Normal joints	scAAVIL-1ra	6 months	*n* = 6	5 × 10^12^ vg	35–50 ng/ml (mean = 40 ng/ml)
					5 × 10^11^ vg	10–30 ng/ml
					5 × 10^10^ vg	5–8 ng/ml (mean = 6 ng/ml)
Watson-Levings 2018 [39]	OCF model	scAAVIL-1ra	10 weeks	*n* = 20	5 × 10^12^ vg	50–200 ng/ml
Goodrich, 2016 [33]	OCF model	scAAVIL-1ra	12 weeks	*n* = 16	5 × 10^12^ vg	790–6400 ng/ml

We observed a numerical decline in PGE_2_ levels with increasing viral dose in this study. PGE_2_ is a well-established and well-characterized mediator of inflammation and synovial fluid levels of PGE_2_ has been associated with clinical pain [53, 54]. We postulate that the low PGE_2_ levels and high IL-1ra levels in the IL-1ra treated joints could be due to the binding of IL-1ra to its receptors thus preventing the IL-1β mediated inflammatory cascade [54].

One of the limitations of this study is the small sample size typical of dosing studies. The PTOA model used in this study is well-established and has been used with consistent results in multiple preclinical trials [5, 29, 30, 55, 56]. Therefore, even with the small sample size, the observations from this study showed a decrease in IL-1ra levels with viral dose that lends confidence in the utility of the results to inform dosing of clinical trials to evaluate efficacy.

As discussed above, the intra-articular IL-1ra levels measured in this scAAVIL-1ra treated PTOA model surpassed those seen in normal joints treated with scAAVIL-1ra. Histopathological examination of articular cartilage revealed higher scores (worsening) with a decrease in vector dose, with the greatest treatment effects observed in chondrone formation subscores. Improvements in scores were observed in the highest and second highest doses tested. Treatment with increasing doses of scAAVIL-1ra was also associated with an improvement in articular cartilage quality based on proteoglycan content and SOFG stain uptake scores. However, the SOFG scores did not vary between the highest and second highest scores suggesting a ceiling effect at the second dose tested (5 x 10^11^vg) such that additional increase in vector dose is unlikely to provide a significant improvement in outcome measures.

Mean IL-1ra levels (263 ng/ml) over 70 days in the joint dosed with 5 x 10^11^vg scAAVIL-1ra in our study were comparable to the levels produced at higher doses in normal and PTOA joints by others [39] (Table 1). Lee et al. previously showed differential localization and persistence of AAV injected into injured joints compared to uninjured joints [57, 58] and thus it was important that this dosing study was completed using our intended surgically induced PTOA model. Interestingly, the ceiling vector dose identified by Watson-Levings et al. [38] of 5 x 10^12^vg when tested in this study showed perivascular cuffing in the synovium. We showed similar chondroprotective effects of the 5 x 10^12^vg using a 10-fold lower dose of 5 x 10^11^vg. Importantly, this dose of 5 x 10^11^vg did not elicit negative synovial reaction in the synovium as observed histologically. This finding suggests that a lower dose of 5 x 10^11^vg scAAVIL-1ra in an injured joint acheives therapeutic levels of IL-1ra with lower risks for adverse joint tissue effects. This is a novel and valuable finding to consider when designing preclinical and clinical studies using this vector for OA therapy as dose optimization will lead to maximizing the therapeutic effects and minimizing any undesirable effects in the synovium as well as a more potentially affordable therapeutic since a 10 fold lower dose allows a reduced vector dose which has important financial implications and decreases the burden of expansive scAAV vector propagation.

## Conclusions

The results of our study suggest that scAAVIL-1ra gene therapy in this equine model of PTOA resulted in an increase in levels of IL-1ra protein. An important finding from this study was that of perivascular lymphocytic infiltration in the synovium at the dose of 5 x 10^12^vg previously established as a ceiling dose that predictably delivered therapeutic levels of protein. Importantly, chondroprotective effects and therapeutic levels of IL-1ra were maintained without observation of this adverse tissue reaction using a 10-fold lower dose of 5 x 10^11^vg scAAVIL-1ra. Therefore, the authors conclude that a lower dose of 5 x 10^11^vg scAAVIL-1ra offer therapeutic effects at lower risk in an injured joint due to the likely upregulation of the CMV promoter in an inflammatory environment within the PTOA joint.

### Supplementary information

Supplemental Table 1

Supplemental Table 2

Supplemental Table 3

## Data Availability

All data generated or analysed during this study are included in this published article.
